# TLRs-JNK/ NF-κB Pathway Underlies the Protective Effect of the Sulfide Salt Against Liver Toxicity

**DOI:** 10.3389/fphar.2022.850066

**Published:** 2022-04-20

**Authors:** Rania Abdel-latif, Gehan Hussein Heeba, Soha Osama Hassanin, Shaimaa Waz, Amr Amin

**Affiliations:** ^1^ Department of Pharmacology and Toxicology, Faculty of Pharmacy, Minia University, El-Minia, Egypt; ^2^ Department of Biochemistry, Faculty of Pharmacy, MTI University, Cairo, Egypt; ^3^ Department of Biochemistry, Faculty of Pharmacy, Minia University, El-Minia, Egypt; ^4^ The College, The University of Chicago, Chicago, IL, United States; ^5^ Department of Biology, UAE University, Al Ain, United Arab Emirates

**Keywords:** hydrogen sulfide, hepatotoxicity, oxidative stress, inflammatory response, toll-like receptors

## Abstract

Hydrogen sulfide (H_2_S) is an endogenously gas transmitter signaling molecule with known antioxidant, anti-inflammatory, and cytoprotective properties. Although accumulating evidence shows the therapeutic potential of H_2_S in various hepatic diseases, its role in cyclophosphamide (CP)-induced hepatotoxicity remains elusive. The present study was undertaken to investigate the impact of endogenous and exogenous H_2_S on toll-like receptors (TLRs)-mediated inflammatory response and apoptosis in CP-induced hepatotoxicity. Either an H_2_S donor (NaHS (100 μM/kg) or an H2S blocker [dl-propargylglycine (PAG) (30 mg/kg, i. p.)], was administered for 10 days before a single ip injection of CP (200 mg/kg). NaHS attenuated conferred hepatoprotection against CP-induced toxicity, significantly decreasing serum hepatic function tests and improving hepatic histopathology. Additionally, NaHS-treated rats exhibited antioxidant activity in liver tissues compared with the CP group. The upregulated hepatic levels of TLR2/4 and their downstream signaling molecules including c-Jun N-terminal kinase (JNK) and nuclear factor-kappa B (NF-κB) were also suppressed by NaHS protective treatment. NaHS showed anti-inflammatory and antiapoptotic effects; reducing hepatic level tumor necrosis factor-alpha (TNF-α) and caspase-3 expression. Interestingly, the cytotoxic events induced in CP-treated rats were not significantly altered upon the blocking of endogenous H_2_S. Taken together, the present study suggested that exogenously applied H_2_S rather than the endogenously generated H_2_S, displayed a hepatoprotective effect against CP-induced hepatotoxicity that might be mediated by TLRs-JNK/NF-κB pathways.

## Graphical Abstract

The proposed schematic diagram illustrates a protective mechanism of NaHS (H2S donor) against CP-induced nephrotoxicity. ROS, reactive oxygen species; TLR2/4; Toll-like receptor-2 and Toll-like receptor 4, NF-κB, nuclear factor kappa-B, JNK; Jun N-terminal kinase, TNF-α; tumor necrosis factor-alpha. 
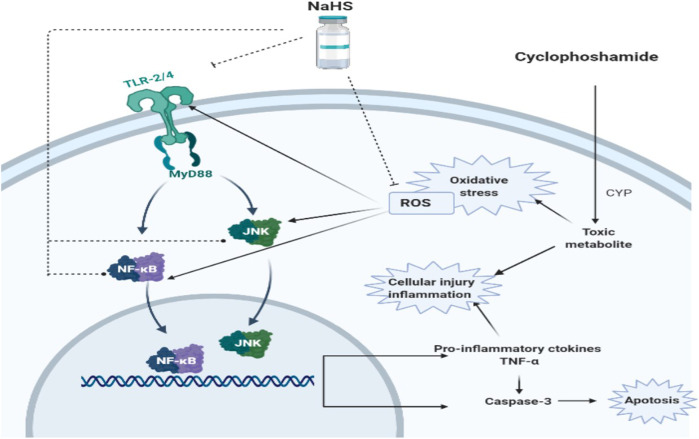

## Introduction

Hydrogen sulfide (H_2_S) is a well-known gas transmitter that mediates various physiology and signaling in various human tissues (Li et al., 2011). The liver is an important site for endogenous H_2_S production that is mediated by cystathionine β-synthase (CBS) and cystathionine γ-lyase (CSE) which is the primary enzyme for H_2_S generation in the liver tissues (Fiorucci et al., 2005; Mustafa et al., 2009). Multiple studies have shown that H_2_S displays a significant role in regulating hepatic physiology and pathology (Li X. et al., 2019; Wu et al., 2019). Accumulated data pointed that the protective effect mediated by H_2_S in the hepatic pathology and toxicity is mainly orchestrated by it is anti-oxidative, anti-inflammatory effects (Fouad et al., 2020; Liu et al., 2020).

Hepatotoxicity is considered one of the major side effects that limit the clinical use of cyclophosphamide (CP) as a potent alkylating agent against various human malignancies and immunological disorders (Taslimi et al., 2019). The hepatotoxicity associated with CP therapy is mainly attributed to the major toxic acrolein produced as a result of CP hepatic bioactivation (King and Perry, 2001; Mahmoud et al., 2017). Acrolein depletes cellular antioxidant defenses and increases the generation of reactive oxygen species (ROS) and oxidative stress in hepatocytes (Mohammad et al., 2012). Consequently, it is currently believed that oxidative stress might represent the main driver of hepatotoxicity associated with CP therapy (Mahmoud et al., 2017; ALHaithloul et al., 2019).

ROS triggers the activation of critical signaling molecules including toll-like receptors (TLRs) which have a critical role in regulating innate immunity and inflammatory responses (Li et al., 2014; Li Y. et al., 2019). TLRs are expressed on different hepatocytes specially Kupffer cells and their activity is strongly correlated to the hepatic stress reaction (Gustot et al., 2006; Ojaniemi et al., 2006). Moreover, TLRs ligation is associated with initiating proinflammatory pathways resulting in activation of both c-Jun N-terminal kinase (JNK) and nuclear factor-kappa B (NF-κB) which have a prominent role in cellular apoptosis (Li et al., 2020). Although TLRs-mediated signals have been implicated in various liver diseases, the significance of TLRs activation and their two different downstream pathways in CP-induced hepatotoxicity are yet to be evaluated.

Previous reports illustrated the therapeutic potential of H_2_S in several hepatic diseases, including hepatic ischemia/reperfusion (I/R) injury (Kang et al., 2009), nonalcoholic steatohepatitis (Li et al., 2018), liver fibrosis (Song et al., 2015), and liver cancer (Yin et al., 2012), But in the term of CP-induced hepatotoxicity, the role of H_2_S is still unclear and needs more investigation. Our recent study showed that H_2_S can protect renal cells against CP-induced oxidative damage (Waz et al., 2021). Additionally, earlier reports showed that H_2_S and CSE biosynthesis during inflammation have displayed TLR/NF-κB and TLR/JNK-dependent manner (Kandil et al., 2010; Zheng et al., 2013; Huang et al., 2016). In this regard, the current study aims to investigate the impact of endogenous and exogenous H_2_S on TLRs pathways in CP-induced hepatotoxicity. A pre-treatment with either NaHS, an exogenous H_2_S donor, or DL-propargylglycine (PAG), an irreversible CSE inhibitor, was investigated in CP-treated rats with hepatotoxicity in terms of modulation of TLRs-mediated inflammatory response, oxidative stress, and apoptosis.

## Materials and Methods

### Drugs and Chemicals

CP (Endoxan®) was purchased from Baxter Oncology GmbH (Germany). NaHS and PAG were purchased from Sigma-Aldrich (St. Louis, MO, United States) and were freshly dissolved in physiological saline upon usage. Polyclonal Rabbit/anti-rat primary antibodies against caspase-3, NF-κB were purchased from Thermo Fischer Scientific Inc./Lab Vision (Fermont, CA, United States). While, TLR4 and JNK (D-2) mouse monoclonal antibodies were purchased from Santa Cruz Biotechnology (CA, United States) and Abcam (MA, United States), respectively. All other chemicals were of the highest available commercial grade.

### Animals and Experimental Design

All experimental procedures were performed in accordance with the international policies (Guide for Care and Use of Laboratory Animals published by the US National Institute of Health; NIH Publication No. 85–23, revised 1996) and approved by the Animal Care Community, Minia University, Egypt (Permit Number: MPH-02-20).

Wistar male rats, 220–240 g body weight (purchased from National research center, Giza, Egypt) were kept at a temperature of 25 ± 2°C, a humidity of 45 ± 5%, and a 12 h light-dark cycle. For accommodation, rats were housed at the faculty of Pharmacy, Minia University, Egypt, for 1 week and allowed free access to standard pellet chow and tap water. After the adaptation period, animals were divided randomly into 4 groups each of 6 rats. Group 1, is a controlled group that received vehicle only. Group 2 (CP group); received only a single dose of 200 mg/kg, i. p on the 11th day of the experiment. The third group (NaHS group); received 100 μM/kg/day for 10 days and a single dose of 200 mg/kg of CP on the 11th day of the experiment. The last group (PAG group); received 30 mg/kg/day for 10 days and a single dose of 200 mg/kg of CP on the 11th day of the experiment. All does were selected based on our preliminary experiments and according to previous studies (Zanardo et al., 2006; Helmy et al., 2019).

### Tissue Sampling and Biochemical Assessment

Euthanasia of rats by urethane (1 g/kg, i. p.) was performed 24 h following the CP injection. Blood was collected *via* decapitation, centrifuged at 1,957×g for 10 min. Serum samples were collected and used for measuring liver function markers of alanine aminotransferase (ALT) and aspartate aminotransferase (AST) using a kinetic kit (BioMed diagnostic, Hannover, Germany).

The liver of each rat was dissected and homogenized in cold potassium phosphate buffer (pH 7.4, 0.05 M). The homogenates were centrifuged at 10,000×g for 10 min at 4°C, and supernatant of each sample was collected to determine lipid peroxides by estimating the hepatic content of thiobarbituric acid reactive substances (TBARS) using 1,1,3,3-tetramethoxypropane as standard (Buege and Aust, 1978). Moreover, hepatic nitric oxide (NO) level was measured as total nitrite/nitrate, using copperized cadmium to reduce nitrate into nitrite, followed by color development with Griess reagent in the acidic medium (Sastry et al., 2002). Hepatic content of reduced glutathione (GSH) was also measured in the collected supernatant using commercially available kits, following the instructions of the manufacturer (Biodiagnostic, Egypt).

ELISA kits from Biomatik (DE, United States) and Abbexa (Cambridge, United Kingdom) were used to assess TLR2 and tumor necrosis factor-alpha (TNF-α), respectively in the liver homogenate according to the manufacturer instructions.

### Western Blot Analysis

TLR4 and JNK protein expressions were analyzed in liver homogenate using the Western blotting method. In brief, parts of liver tissues were homogenized in lysis buffer (20 mMTris-HCl pH 7.5, 50 mM 2-mercaptoethanol, 5 mM EGTA, 2 mM EDTA, 1% NP40, 0.1% SDS, 0.5% deoxycholic acid, 10 mMNaF, 1 mM PMSF, 25 mg/ml leupeptin, 2 mg/ml aprotinin) and centrifuged at 14,000×g at 4°C for 30 min. Aliquots containing (20 μg/lane) total protein were boiled with an equal volume of 2× Laemmli sample buffer containing 10% 2-mercaptoethanol, 20% glycerol, 4% SDS, 0.004% bromophenol blue, and 0.125 M TrisHCl. The aliquots were then loaded onto a 10% polyacrylamide gel (SDS-PAGE) for protein separation. After electrophoresis, the gels were transferred to PVDF membrane. To reduce background staining, the membranes were incubated in tris-buffered saline with 0.1% Tween 20 (TBST) buffer and 3% bovine serum albumin (BSA) at room temperature for 1 h. Then, membranes were incubated with primary antibodies of TLR4 (Catalog # sc-293072 AF790, Dilution 1:200), JNK (D-2) (Catalog # sc-7345 AF790, dilution 1:200) in a non-fat milk/PBS buffer overnight at 4°C. The membranes were washed extensively and then incubated with a secondary antibody conjugated to Goat anti-rabbit IgG horseradish peroxidase (Novus Biologicals, United States) for 1 h. Protein bands were detected by a standard enhanced chemiluminescence method (ClarityTM Western ECL substrate Bio-Rad, Catalog # 170–5060). The chemiluminescent signals were captured using a CCD camera-based imager and densitometry measurements were made using ChemiDoc MP Imager. The densities of target protein bands were normalized to the corresponding density of the β-actin band and presented as a ratio of the relative optical density (ROD).

### Histopathological Examination

Parts of liver tissues were fixed in 10% neutral buffered formalin (24–72 h), dehydrated, then embedded in paraffin cubes. Sections were cut at 4 μm, stained with hematoxylin and eosin, and examined under a light microscope by a specialist unaware of the slide identity. Additionally, a semiquantitative score was used to assess the percent of histopathological alterations of total fields examined as follows; 0: absent; 1: mild, <25% of hepatic tissue affected; 2: moderate, <26–50% of hepatic tissue affected; 3: severe, <50% of hepatic tissue affected.

### Immunohistochemical Analysis

According to a previously described method (Maae et al., 2011), 4 μm hepatic sections were dewaxed and rehydrated through a graded series of ethanol and rinsed in water. Sections were mounted in 33% hydrogen peroxidase for 5 min to block endogenous peroxidase activity. To block non-specific binding, Ultra V block was also used. Sections were incubated with the primary antibodies of NF-κB (Catalog # RB-1638-P0, dilution 1:100) or caspase-3 (Catalog # RB-1197-R7, dilution 1:100) overnight in a humid chamber at 4°C. For detection, a secondary antibody HRP Envision kit (DAKO) was added for 20 min followed by visualization with diaminobenzidine (DAB) chromogen for 15 min for the development of the color reaction. Finally, slides were counterstained with Mayer’s hematoxylin, dehydrated, and slipped covered for microscopic examination. Semi-quantitative analysis was performed for each sample by determining area % of immunoexpression levels of caspase 3 and NF-κB in six randomly selected fields within each image. All measurements and analyzed data were obtained using a full HD microscopic imaging system operated by Leica Application module for Histological analysis (Leica Microsystems GmbH, Germany).

### Statistical Analysis

Results were expressed as means ± SEM. The statistical significance was assessed using a one-way analysis of variance (ANOVA) followed by Tukey–Kramer post-analysis test for comparison between groups with normal distribution. A Kruskal–Wallis test was used for the abnormal distribution of data then Dunn’s Multiple Comparison test was applied. *p* < 0.05 was considered statistically significant. GraphPad^®^ Prism (version 8.0.2) was used for statistical calculations (SanDiego, CA, United States).

## Results

### Effect on Liver Function Parameters

**FIGURE 1:**
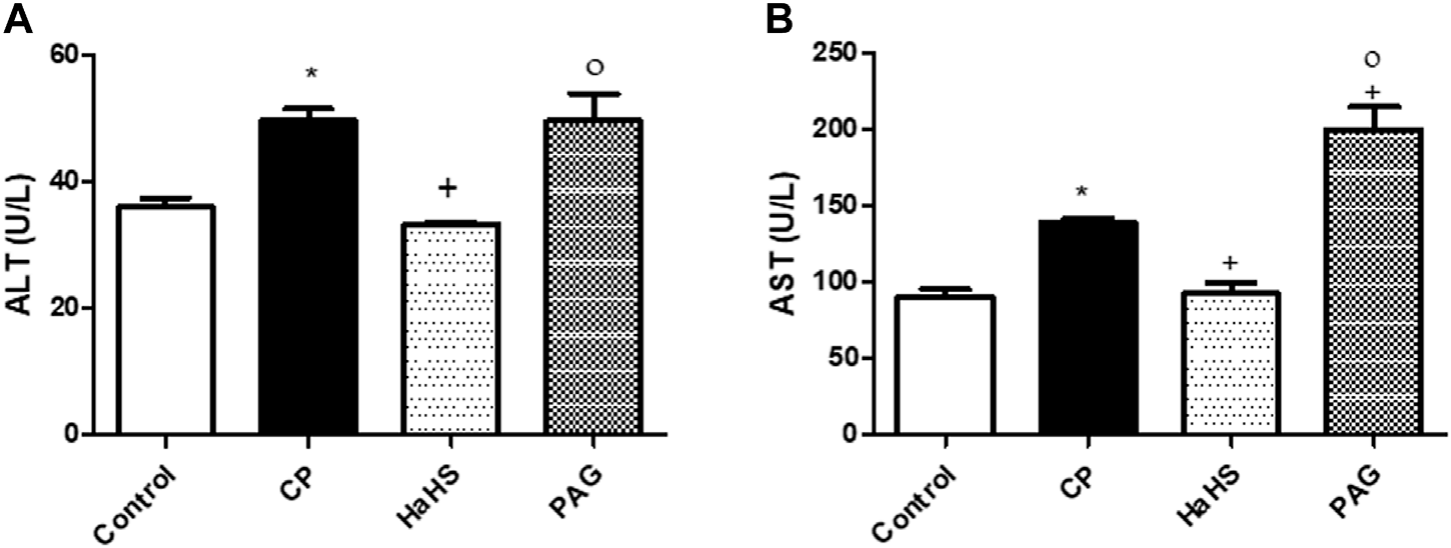
Effect of NaHS and PAG on the serum levels of ALT **(A)** and AST **(B)** in CP-induced induced hepatotoxicity in rats. Data are represented as mean ± SEM. *,+,° are significantly different from control, CP and NaHS groups, respectively, where n = 6 and *p* < 0.05. CP; cyclophosphamide,PAG; DL-propargylglycine, ALT; alanine aminotransferase, AST; aspartate aminotransferase.

As presented in Figure 1, administration of a single CP dose (200 mg/kg, i. p.) significantly elevated (*p* < 0.05) both serum ALT (Figure 1A) and AST levels (Figure 1B) compared to the control group. Administration of NaHS for consecutive 10 days before CP treatment conferred protection against elevated serum ALT and AST levels. Well noted, serum ALT and AST levels showed a significant decrease in the NaHS group compared to the CP group (33.24 ± 0.40 and 92.94 ± 6.494 vs. 49.57 ± 1.89 and 138.8 ± 3.563 U/L, respectively). Interestingly, levels of serum ALT did not change significantly in the PAG group compared to the CP group. However, serum levels of ALT and AST in the NaHS group have significantly decreased compared to that of the PAG group (92.94 ± 6.49 vs. 138.8 ± 3.56 U/L, *p* < 0.05).

### Effect on Hepatic Histopathological Changes

**FIGURE 2:**
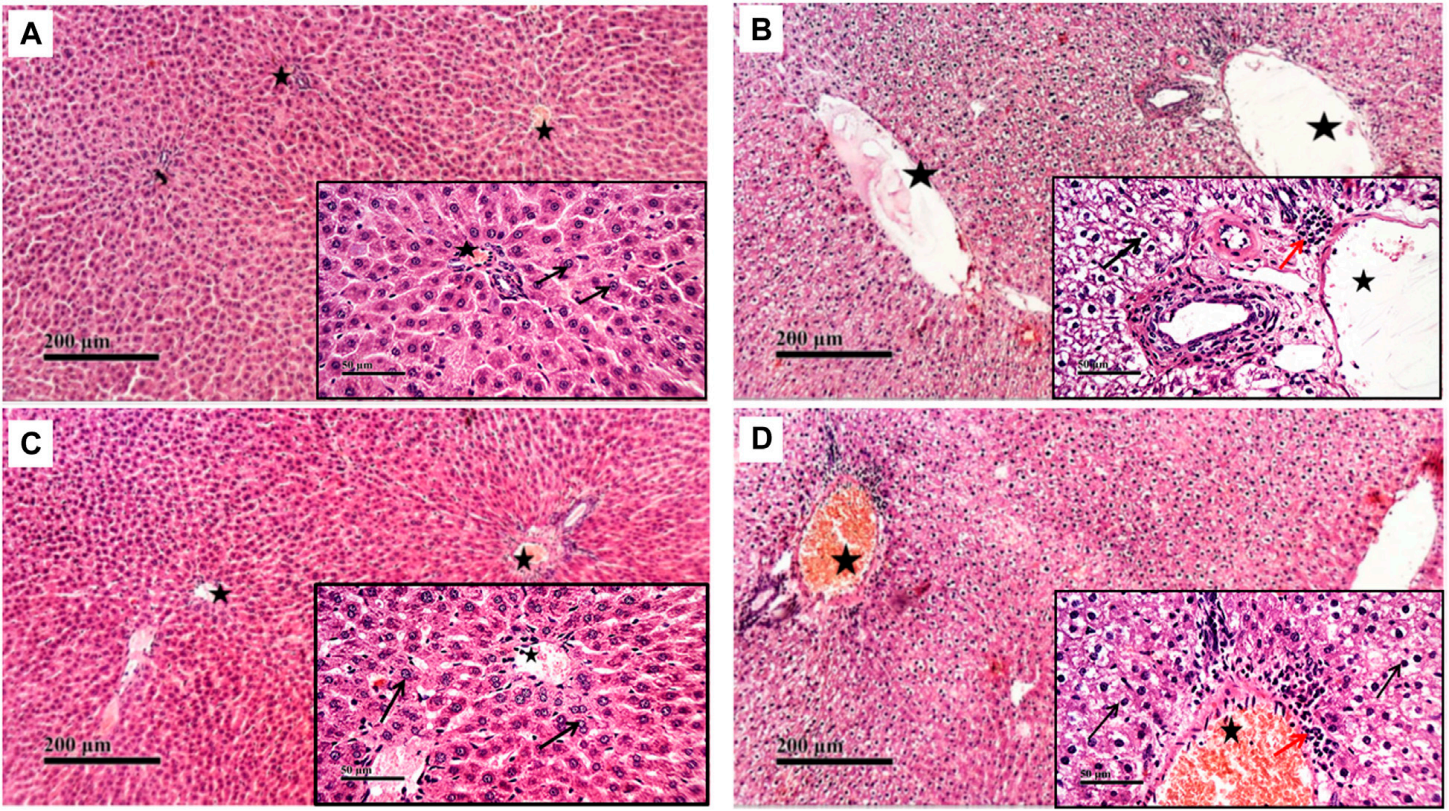
Effect of NaHS and PAG on rats’ liver tissues stained with hematoxylin and eosin (H&E) in CP- induced hepatotoxicity (×200 and ×400). Liver tissue of control group **(A)** showed normal morphological features of hepatic parenchyma with many apparent intact radiating hepatocytes. Liver tissues of rats treated with CP **(B)** showed sever diffuse hepatocellular vacuolar degeneration with karyopyknosis (arrow) accompanied with moderate dilatation of hepatic blood vessels (star) and mild records of periportal inflammatory cells infiltrates (red arrow). Liver tissues of NaHS rat group) showing intact histological structure of hepatic lobule with few degenerative changes (black arrow) mild hepatic blood vessel dilatation (star) or inflammatory cells infiltrates **(C)**. microscopic examination of liver tissues of PAG rat group **(D)** showed wide diffuse areas of vacuolar degenerative changes of most of hepatocytes (arrow) with many dilated, congested hepatic blood vessels (star) and mild records of focal perivascular inflammatory cells infiltrates (red arrow).

**FIGURE 3:**
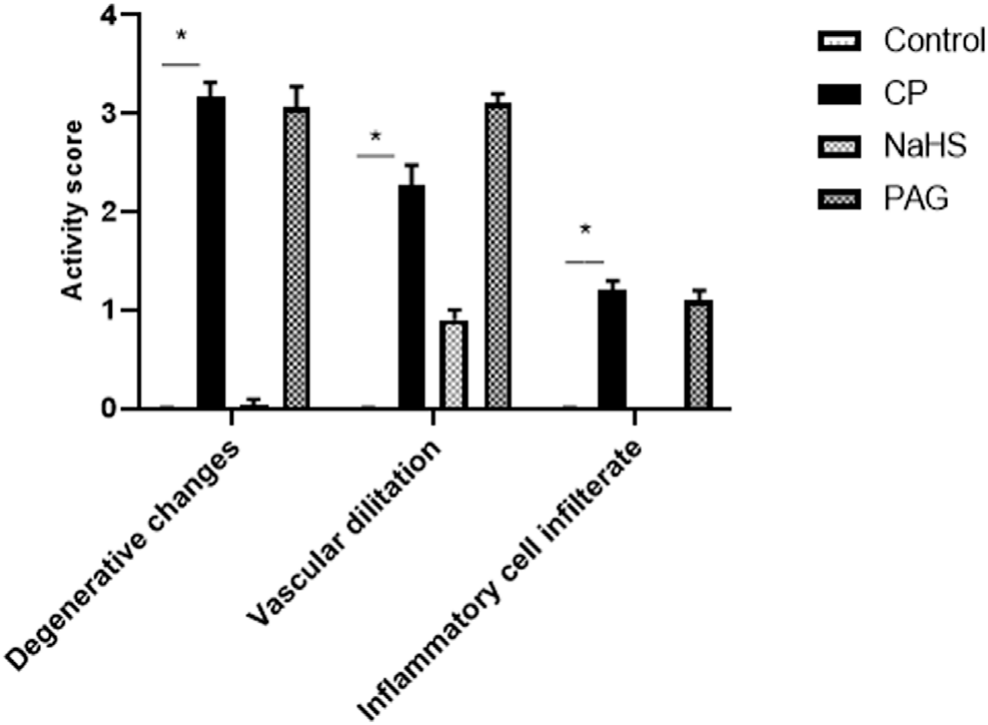
Effect of NaHS and PAG on the severity of histopathological lesions in CP-induced hepatotoxicity in rats. All parameters were represented as mean score. Kruskal–Wallis and then Dunn’s test was applied for comparison. * is significantly different from control where *p* < 0.05. CP; cyclophosphamide, PAG; DL-propargylglycine.

Compared to the normal histological feature exhibited in the control group, rats treated with CP showed severe histological changes in all hepatic lobular zones in form of vacuolar along with many figures of karyopknosis, as well as periportal inflammatory cells infiltration. Hepatic tissues of the NaHS-treated rats showed well-protected and better organized histological features of hepatic parenchyma with minimal records of degenerative changes or inflammatory cell infiltrates compared to hepatic tissues of CP-treated rats. Comparable to the CP group, liver tissues of the PAG group showed severe degenerative changes in terms of congested blood vessels and severe inflammatory cell infiltrate (Figures 2, 3).

### Effect on Hepatic Oxidant Status

A dramatic depletion of the non-enzymatic \was observed in liver tissues of rats treated with CP as compared to control rats. The hepatic GSH content was replenished by 17 and 13% in CP-treated rats protected by NaHS administration as compared to the non-treated CP group and PAG group, respectively. On the other hand, hepatic GSH content in the PAG group did not change significantly compared to the CP group.

In parallel, a profound spike in hepatic TBARS content (64.71%), a hallmark of lipid peroxidation, occurred in the CP group as compared to the control group. Compared to the CP group, hepatic TBARS was mitigated by NaHS pretreatment (62.55 ± 3.04 vs. 96.33 ± 1.54 nmol/g tissue), while TBARS showed a non-significant increment along with PAG pretreatment compared to the CP group (102.7 ± 3.71 vs. 96.33 ± 1.54 nmol/g tissue). Likewise, CP-treated rats exhibited pronounced elevation in NOx content by 1.65 folds compared to the control group. NaHS pretreatment for 10 days led to a significant decrease in NOx level in CP-treated rats (6.29 ± 0.54 vs. 11.62 ± 0.74 nmol/g tissue). Unlikely. PAG pretreatment for 10 successive in CP-treated rats resulted in a significant increase in hepatic NOx content compared to the CP group (15.33 ± 1.34 vs. 11.62 ± 0.74 nmol/g tissue) (Table 1).

**TABLE 1:** Effects of NaHS and PAG in CP-induced hepatotoxicity in rats.

Groups	GSH (mg/g Tissue)	TBARS (nmol/g Tissue)	NOx (nmol/g Tissue)
Control	0.822 ± 0.011	52.2 ± 1.4	4.38 ± 0.32
CP	0.633 ± 0.018*	96.33 ± 1.54*	11.62 ± 0.74*
NaHS	0.7410 ± 0.023 +	62.55 ± 3.04 +	6.29 ± 0.54 +
PAG	0.653 ± 0.015°	102.7 ± 3.71°	15.33 ± 1.34 +°

Data are represented as mean ± SEM. *,+,° Significantly different from control, CP, and NaHS, groups, respectively; where n = 6 and *p* < 0.05.CP; cyclophosphamide, PAG; DL-propargylglycine, GSH; reduced glutathione, TBARS; thiobarbituric acid reactive substances, NOx; Total nitrite/nitrate.

### Effect on TLR4 and JNK Protein Expressions

**FIGURE 4:**
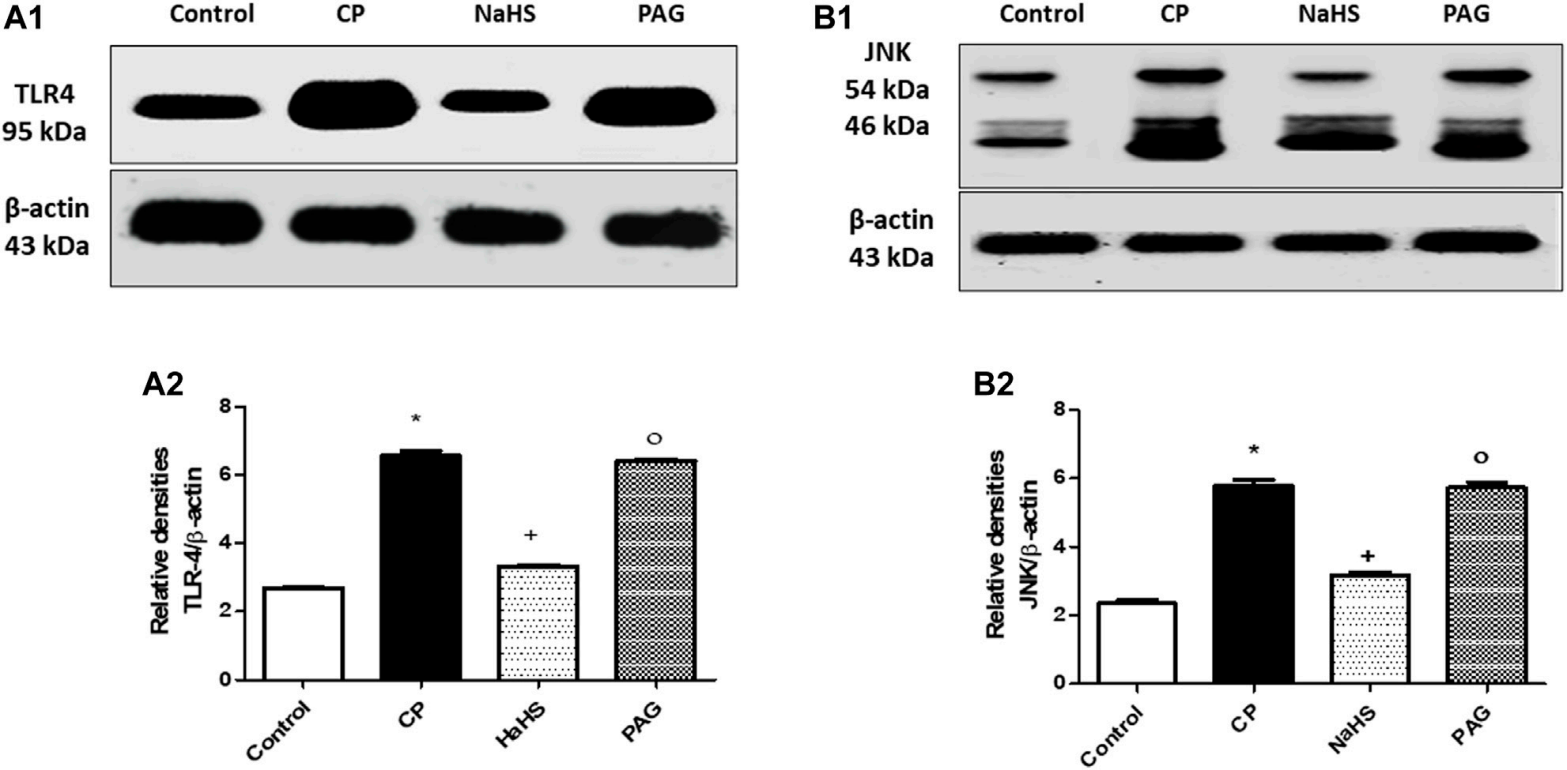
Representative Western blot analysis of the effect of NaHS and PAG on hepatic TLR4 **(A)** and p-JNK **(B)** protein expressions in CP-induced hepatotoxicity in rats, showing protein bands of each group (upper panel) and graphs present their densitometric analysis (lower panel). Data are represented as mean ± SEM. *,+,° are significantly different from control, CP and NaHS groups, respectively, where n = 6 and *p*<0.05. CP; cyclophosphamide, PAG; DL-propargylglycine, TLR4; Toll-like receptor4, p-JNK; phosphorylated Jun N-terminal kinase.

Hepatic protein expression of both TLR4 (Figure 4A) and JNK (Figure 4B) were significantly elevated almost by 1.44 and 1.45-fold, respectively in CP-treated rats compared to their respective control groups. Administration of NaHS for 10 days prior to CP treatment showed a significant reduction in the hepatic protein expression of both TLR4 (44.92%) and p-JNK (44.3%) compared to their respective CP groups. Hepatic protein expression of both TLR4 and JNK did not change significantly in CP-treated rats upon PAG pretreatment compared to the CP group. Meanwhile, significant decreases are observed in both hepatic TLR4 and JNK expressions in the NaHS group compared to the PAG group.

### Effect on Hepatic Levels of TLR2 and TNF-α

**FIGURE 5:**
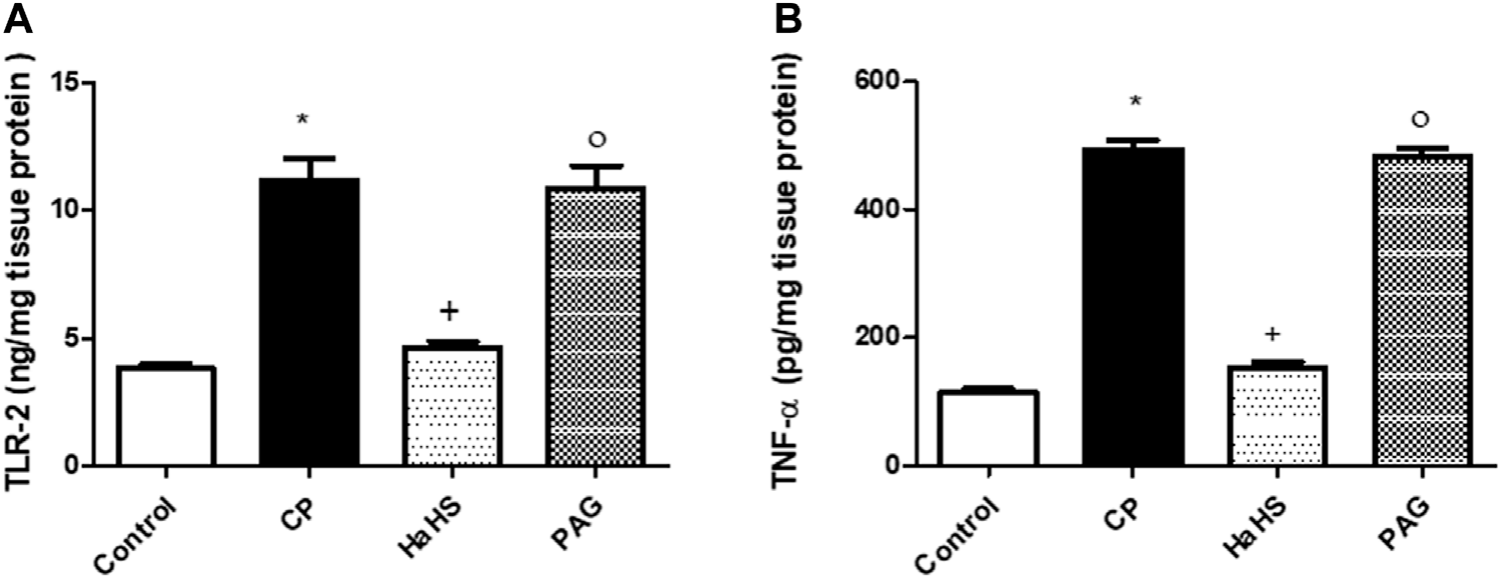
The effect of NaHS and PAG on hepatic level of TLR-2 **(A)** and TNF-α **(B)** in CP-induced hepatotoxicity in rats. Data are represented as mean ± SEM. *,+,° are significantly different from control, CP and NaHS groups, respectively, where n = 6 and *p*<0.05. CP; cyclophosphamide, PAG; DL-propargylglycine, TLR2; Toll-like receptor 2, TNF-α; tumor necrosis factor-alpha.

Figure 4 depicts ELISA measures for TLR2 (Figure 5A) and TNF-α (Figure 5B) levels in the liver tissues of the different study groups. CP-treated rats showed a significant elevation in hepatic levels of TLR2 and TNF-α compared to control groups. Pretreatment with NaHS markedly mitigates both TLR2 and TNF-α elevation observed in CP-treated rats, while PAG pretreatments did not show significant changes in the aforementioned measures compared to the CP group. Compared to the PAG group, significant decreases in hepatic levels of both TLR2 and TNF-α were detected in the NaHS group.

### Effect on the Immunostaining of NF-κB and Caspase-3

**FIGURE 6:**
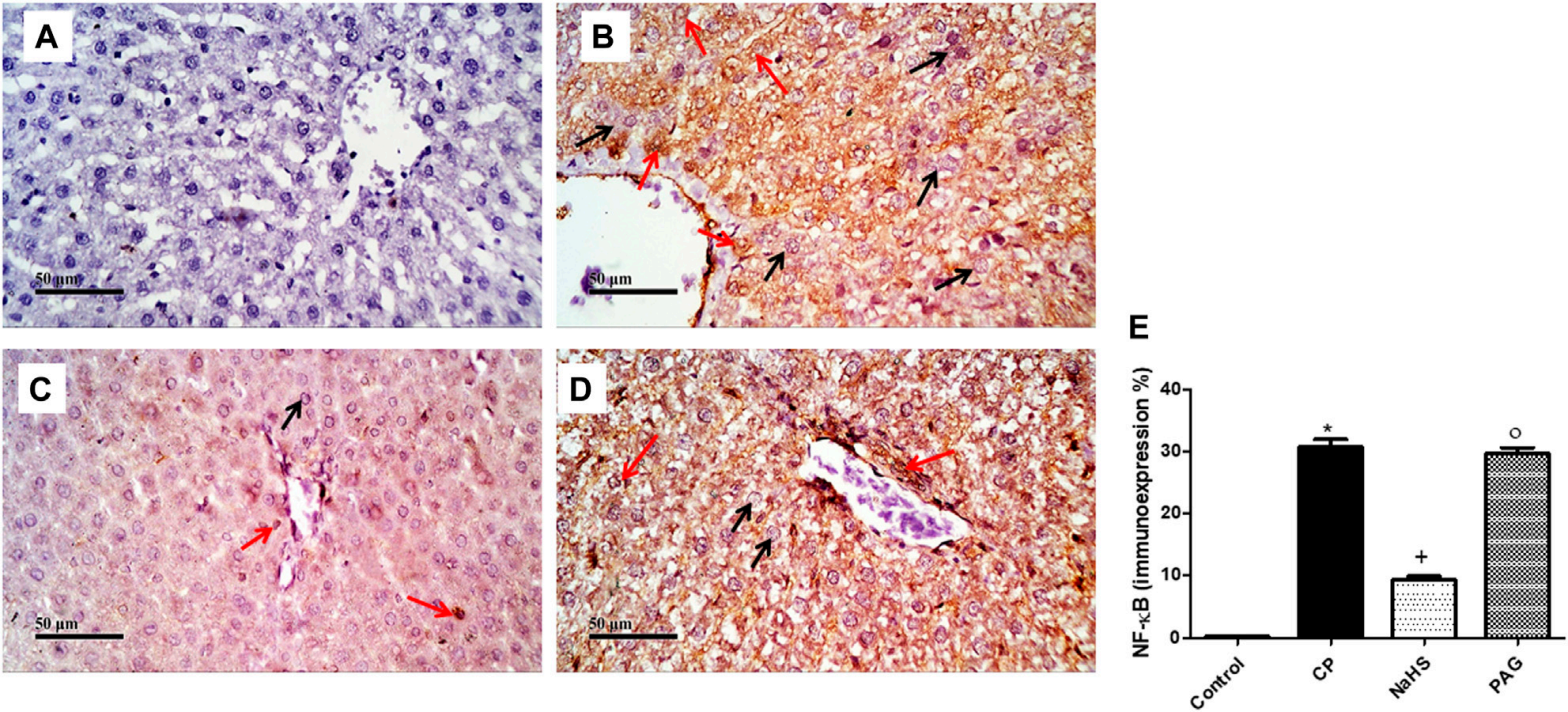
Representative photomicrographs of immunohistochemical analysis of hepatic NF-κB protein expression **(A)** Control group, **(B)** CP group, **(C)** NaHS group, **(D)** and PAG group. All reactive hepatocytes are labeled with red arrows, while black arrows indicate negative reactive hepatocytes. **(E)** A semi-quantitative analysis of NF-κB in rat’s liver tissue. Data are represented as mean ± SEM. *, +, ° are significantly different from control, CP and NaHS groups, respectively, where n = 6 and *p* < 0.05. CP; cyclophosphamide, PAG; DL-propargylglycine, NF-κB; nuclear factor kappa B.

**FIGURE 7:**
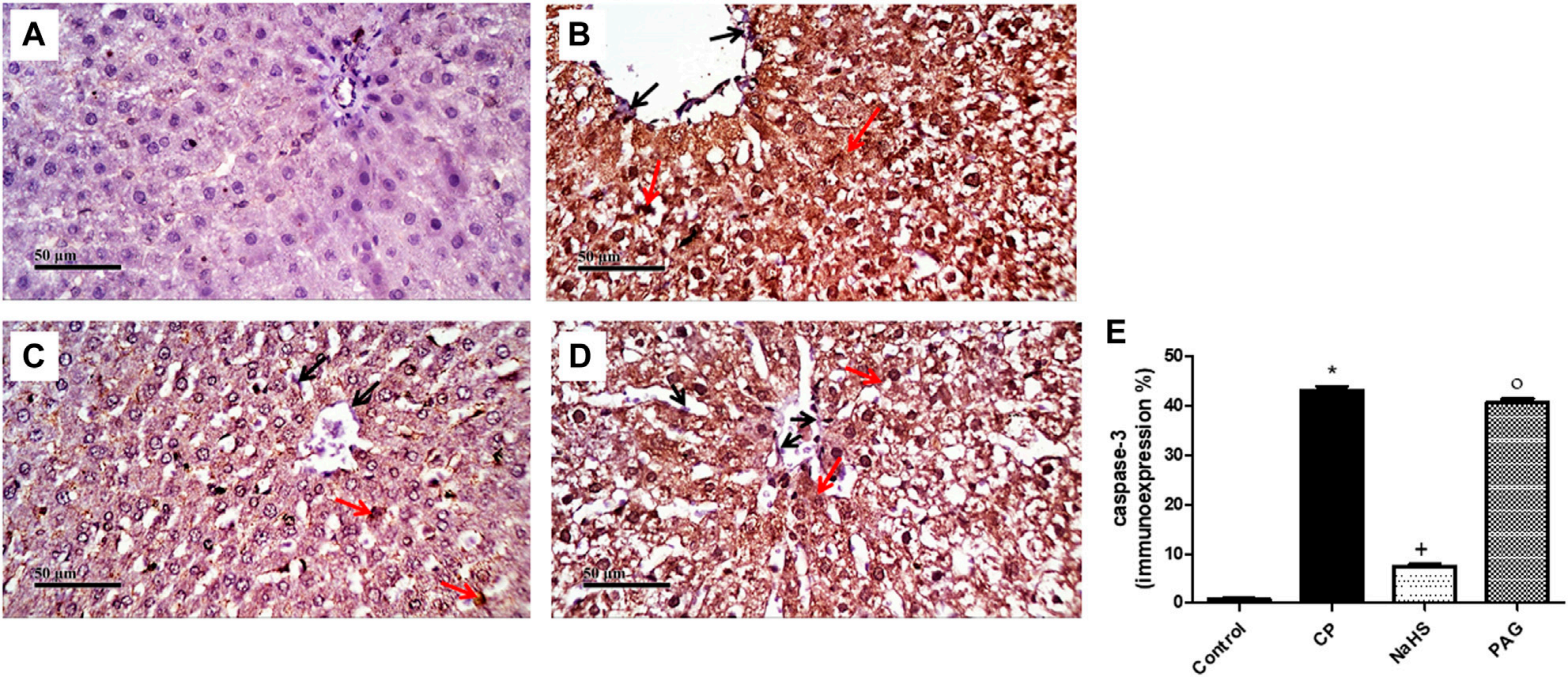
Representative photomicrographs of immunohistochemical analysis of hepatic caspase-3 protein expression **(A)** Control group, **(B)** CP group, **(C)** NaHS group, and **(D)** PAG group. All reactive hepatocytes are labeled with red arrows, while black arrows indicate negative reactive hepatocytes. **(E)** A semi-quantitative analysis of caspase-3 in rat’s liver tissue. Data are represented as mean ± SEM. *, +, ° are significantly different from control, CP and NaHS groups, respectively, where n = 6 and *p* < 0.05. CP; Cyclophosphamide, PAG; DL-propargylglycine.

CP administration induced a significant (*p* < 0.05) increase in both NF-κB (Figure 6) and caspase-3 (Figure 7) immunoexpression levels in the liver when compared with the control rats. On the contrary, CP-induced rats pre-treated with NaHS showed a significant decrease in hepatic NF-κB levels by 69.61 and 68.39% compared to CP and PAG groups, respectively (Figure 6). Similarly, a significant decrease in hepatic caspase-3 expression was detected in the NaHS group by 82.64 and 81.58% compared to CP and PAG groups, respectively (Figure 7). Worth mentioned, hepatic expression of either NF-κB or caspase-3 did not show any significant changes in the PAG group compared to the CP group.

## Discussion

Hepatotoxicity is one of the major side effects that limit the therapeutic use of CP in the clinical setting. Hepatotoxicity associated with CP antineoplastic action is mainly attributed to acroline, the toxic CP hepatic metabolite, which evolves ROS and highly interferes with the antioxidant defense system. Indeed, oxidative stress is the main detrimental factor that has been formerly incriminated in the pathogenesis of liver injury and dysfunction induced by CP. Findings from the present study showed that CP-treated rats showed a liver injury evident by altered hepatic histological structure and increased serum liver markers. The observed liver injury in CP-treated rats is associated with increased oxidative stress in terms of increased MDA and NO_x_^−^ levels along with decreased GSH levels in the liver.

Accumulating evidence supported the beneficial effect of H_2_S-based therapy in different models of liver injury (Kang et al., 2009; Zhang et al., 2013; Wu et al., 2015; Wu et al., 2019; Fouad et al., 2020). Current data showed that pretreatment with H_2_S donor (NaHS) suppressed lipid peroxidation and elevated levels of GSH in liver tissues of CP-induced rats. The potent anti-oxidant effect that is recorded in the NaHS group went along the marked improvement in liver function and pathological changes. The current results are consistent with previous reports showed H2S regulation of redox reaction and exert ROS scavenger effect (Mani et al., 2011; Huang et al., 2016). Lee et al., 2014) showed that activation of cysteine/cystine transporters mediated by H2S leads to elevating GSH production which protects against ROS-mediated damage.

Interestingly, pretreatment with PAG, an irreversible inhibitor of CSE, in CP-injured rats did not show any alterations in liver function, structure, or oxidative status compared to CP-treated animals. Although hepatic expression of CSE exceeds that of CBS by nearly 60 folds (Singh and Banerjee, 2011), a study by Mani et al. (2011) showed that CSE knockouts were not associated with any indices of hepatic disorders including ALT, AST, and albumin levels. The neutral effect of CSE inhibition in CP-treated animals on hepatic function could be justified by the insignificant role of CSE on oxidative status. Previous results showed that increased plasma homocysteine levels are associated with CBS deletion in the liver but not CSE deletion (Robert et al., 2005; Yang et al., 2008). As elevated homocysteine plasma triggers ROS production and impairs GSH-related anti-oxidant defense (Liu et al., 2013), reduced hepatic oxidative stress is suggested to be linked to CBS, not CSE.

In the liver tissues, TLRs are extensively expressed in hepatocytes and other cells, and they showed a critical role in liver physiological function (Schwabe et al., 2006; Chen and Sun, 2011). The most prominent hepatic TLRs are TLR2 and TLR4 have been shown to be important for the production of the inflammatory response observed in different models of experimental hepatic injury such as hepatic ischemia, inflammation, and acute hepatic failure (Tu et al., 2012; Mahmoud et al., 2014; Qiu et al., 2018). Importantly, activation of TLRs signaling pathways is reported to be regulated during oxidative stress (Gustot et al., 2006). Additionally, it was reported that intracellular ROS production is a potential activator for TLR2/4 expressions (Huang et al., 2011). In our data, the hepatic level of TLR2/4 showed a significant increase upon CP-treatment. These elevated levels were detected along with oxidative stress exhibited in this group, which conceivably showed that ROS may be the trigger for TLR4/2 expression. Equally important, previous studies showed that TLR2/4, mediated inflammatory and oxidative stress activities, are initiated in response to damaged cells in the liver tissue (Chang and Toledo-Pereyra, 2012; Mahmoud et al., 2014). Hence, it is suggested that liver injury induced by CP-toxicity may be a direct stimulator for TLR2/4 expression which, in turn, can induce ROS production and causes oxidative stress. However, this hypothesis requires further investigation.

In conjunction with oxidative stress, previous studies reported significant increments of inflammatory cytokines including NF-κB and TNF-α in the serum of CP-treated rats (Mahmoud et al., 2017). It is well known that TLR2/4 induced MyD88-dependent and independent signaling cascade to initiate translocation of NF-κB and subsequent activation of TNF-α (Matsumura et al., 2000; Chang and Toledo-Pereyra, 2012). These findings went along with our results which showed elevated levels of NF-κB and TNF-α in the liver tissues of CP-treated animals along with increased hepatic TLR2/4 detected in the same group.

Data of the present study showed substantial decrease of TLR2/4 and NF-κB expressions in the liver tissue of the NaHS group that could be explained by the ROS quenching effect of exogenous H_2_S. The current data is supported by previous studies showed that H2S exert an anti-inflammatory effect *via* multiple mechanisms including upregulation of antioxidant defense and regulating inflammatory signal transduction (Pan et al., 2011; Li et al., 2016). Hence, it is also plausible to attribute the insignificant change in hepatic TLR2/4 levels observed along with PAG pretreatment to it is neutral effect on the oxidative status of CP-treated rats. Furthermore, the results of (Huang et al., 2016) consolidate the data of the present study as it concluded that exogenous H_2_S can mediate its anti-inflammatory effect *via* a direct suppression of activated TLR4/NF-κB pathway in hyperglycemic-injured cardiomyocytes (Huang et al., 2016).

A recent study by Mohammed et al. (2020) showed the involvement of basic inflammatory pathways such as JNK in an animal model of CP-induced hepatotoxicity (Mohammed et al., 2020). The aforementioned study showed that oxidative and nitrosative stress induced by CP contributes to JNK activation in the liver tissues, which results in piling up the ROS in the cell. Moreover, other previous results showed that JNK is activated by ROS generated as a result of GSH-depleted mitochondria and peroxynitrite formation in liver-injured animal models (Hanawa et al., 2008; Kandil et al., 2010). On the other hand, other studies suggested that TLR2/4-induced JNK stimulates the production of pro-inflammatory cytokines mainly TNF-α, besides ROS which is believed to be a major contributor to liver injury (Chang and Toledo-Pereyra, 2012; Li W. et al., 2019; Gong et al., 2019). Here, we demonstrated an upregulation of JNK expression in CP-injured liver tissues along with increased hepatic TLR2/4 protein levels and oxidative stress. More importantly, the results of the present study showed a significant decrease in hepatic JNK expression along with NaHS pretreated CP-induced injured rats. The accumulated data point to the role of exogenous H_2_S in modulating mitogen-activated protein kinases (MAPKs) signaling and JNK as a dominant effector of MAPKs in liver tissues. In fact, the reported data showed an ameliorating effect on oxidative stress and cell injury along with H_2_S supplement that may be secondary to alleviating MAPK/JNK signaling pathway (Xu et al., 2011; Yuan et al., 2017; Li X. et al., 2019). In harmony, NaHS pretreatment showed protection against oxidative stress in CP-treated animals along with decreased hepatic protein expression of p-JNK. Although CSE is responsible for more than 90% of hepatic biosynthesis capacity (Singh and Banerjee, 2011), it is interesting to find out that hepatic p-JNK expression in the PAG group did not change in comparison to CP-treated rats. Such findings reinforced the probability that endogenous H_2_S may not be adequately abated due to the existence of alternative routes of H_2_S production including the action of CBS and 3-mercaptopyruvate sulfurtransferase (3-MST).

ROS and pro-inflammatory cytokines could potentially trigger apoptosis and cell death in liver tissue (Heeba and Mahmoud, 2014; Hamza et al., 2020). Here, CP treatment induced apoptotic cell death as shown by increased hepatic caspase-3 expression along with enhanced oxidative stress which coincided with previous studies (Caglayan et al., 2018; Aladaileh et al., 2019). The present results showed that CP-mediated excessive ROS levels are associated with activated TLR2/4 signaling pathways which are suggested to have a central role in initiating an apoptotic response. Indeed, it is believed that TLR2/4 can activate the initiator of the apoptotic cascade *via* interaction with the Fas-associated death domain protein (FADD) through MyD-88 which leads to ultimately activated caspase-3 (Li et al., 2014; Liu et al., 2015). The anti-apoptotic effect of H_2_S donner was emphasized in our work *via* detecting a significant reduction of hepatic caspase-3 expression in the NaHS-treated group. In harmony, Tan et al. (2015) reported the anti-apoptotic and anti-inflammatory actions of H_2_S in renal I/R injury *via* ameliorating the activated TLR2/4 (Tan et al., 2015). Conspicuously, inhibiting TLR2/4 signaling averts translocation of NF-κB and activation of JNK which would eventually lead to preventing cell apoptosis (Li et al., 2014). Additionally, another study showed that the anti-apoptotic and anti-inflammatory effects of exogenous H_2_S may be mediated by inhibiting JNK phosphorylation (Li X. et al., 2019), downregulation of NF-κB (Li et al., 2016), or by direct inhibition of ROS production (Spassov et al., 2017). Consequently, it is possible that the neutral effect of PAG pretreatment on hepatic caspase-3 expression might be linked to its non-significant effect on the oxidative status of CP-treated rats.

## Conclusion

Taken together, these findings suggested that exogenous H_2_S plays an important role in the protection against CP-induced hepatotoxicity *via* alleviating oxidative stress, inflammatory and apoptotic responses in liver tissues of CP-treated animals. The present study also indicated that ameliorating ROS generation and suppression of TLRs-JNK/NF-κB signaling pathways are the proposed molecular mechanisms underlying the hepatoprotective effect of NaHS in this model. Further investigations are warranted to explore the clinical application of H2S donors against CP-induced hepatotoxicity.

## Data Availability

The original contributions presented in the study are included in the article/supplementary material, further inquiries can be directed to the corresponding authors.
